# Thiacloprid Induced Developmental Neurotoxicity via ROS-Oxidative Injury and Inflammation in Chicken Embryo: The Possible Attenuating Role of Chicoric and Rosmarinic Acids

**DOI:** 10.3390/biology10111100

**Published:** 2021-10-25

**Authors:** Mayada R. Farag, Samah R. Khalil, Asmaa W. Zaglool, Basma M. Hendam, Amr A. Moustafa, Raffaella Cocco, Alessandro Di Cerbo, Mahmoud Alagawany

**Affiliations:** 1 Forensic Medicine and Toxicology Department, Veterinary Medicine Faculty, Zagazig University, Zagazig 44519, Egypt; resamah@zu.edu.eg; 2 Animal Wealth Development Department, Faculty of Veterinary Medicine, Zagazig University, Zagazig 44519, Egypt; Awawadala@zu.edu.eg; 3 Department of Husbandry and Development of Animal Wealth, Faculty of Veterinary Medicine, Mansoura University, Mansoura 35516, Egypt; asmahendam@mans.edu.eg; 4 Biochemistry Department, Faculty of Veterinary Medicine, Zagazig University, Zagazig 44519, Egypt; amr.moustafa@gmx.de; 5 Department of Veterinary Medicine, University of Sassari, via Vienna 2, 07100 Sassari, Italy; rafco@uniss.it; 6 School of Bioscience and Veterinary Medicine, University of Camerino, 62024 Matelica, Italy; 7 Poultry Department, Faculty of Agriculture, Zagazig University, Zagazig 44519, Egypt

**Keywords:** chicken embryos, thiacloprid, chicoric acid, rosmarinic acid, developmental neurotoxicity

## Abstract

**Simple Summary:**

The current study was designed to evaluate the negative impact of thiacloprid (TH) on the brain tissue of developing chicken embryo models and to evaluate the modulatory effects of chicoric (CA) and rosmarinic (RA) acids. The eggs were injected in ovo with different doses of TH (0.1, 1, 10, and 100 μg/egg). TH significantly increased the oxidative damage in the brain of exposed embryos in a dose-dependent manner (*p* < 0.001). TH significantly elevated the oxidative stress markers; protein carbonyl, malondialdehyde (MDA) content, and DNA damage (*p* < 0.001). Myeloperoxidase (MPO) activity and NO significantly increased with overexpression of the pro-inflammatory cytokines (IFN-γ; interferon gamma, TNF-α; tumor necrosis factor alpha, and IL-1β; interleukin-1 beta), stress-related and apoptotic genes (NF-KB, Caspase-3) in the brain tissue on both a biochemical and molecular levels (*p* < 0.05), while downregulating the expression of antiapoptotic Bcl-2. Co-treatment of CA and RA with TH markedly decreased the insecticide-induced toxicity with a prominent synergistic effect (*p* < 0.05). In conclusion, TH is suggested to be a possible neurotoxic to embryos of vertebrates and possibly humans. The study also revealed the antioxidant, anti-inflammatory, genoprotective, and antiapoptotic properties of CA and RA against TH toxicity.

**Abstract:**

Insecticides are widely employed in agriculture to control pests and as major factors for enhancing crop productivity. Thiacloprid (TH) is one of the most-used insecticides worldwide. In this study, the negative impact of TH on the brain tissue of developing chicken embryo models and the modulatory effect of chicoric (CA) and rosmarinic (RA) acids were investigated. The eggs were injected in ovo with different doses of TH (0.1, 1, 10, and 100 μg/egg). TH significantly increased the oxidative damage in the brain of exposed embryos in a dose-dependent manner (*p* < 0.05). TH significantly elevated the oxidative stress markers; protein carbonyl, malondialdehyde content, and DNA damage (*p* < 0.05). Myeloperoxidase activity and nitric oxide significantly increased with overexpression of the pro-inflammatory cytokines (interferon gamma, tumor necrosis factor alpha, and interleukin-1 beta) and stress-related and apoptotic genes (NF-KB, Caspase-3) in the brain tissue on both biochemical and molecular levels (*p* < 0.05), while downregulating the expression of antiapoptotic Bcl-2. Co-treatment of CA and RA with TH markedly decreased the insecticide-induced toxicity with a prominent synergistic effect (*p* < 0.05). In conclusion, TH is suggested to be a possible neurotoxic to embryos of vertebrates including human. The study also revealed the antioxidant, anti-inflammatory, genoprotective, and antiapoptotic property of CA and RA against TH toxicity.

## 1. Introduction

Insecticides are widely employed in agriculture to control pests and enhance crop productivity. However, the extensive and uncontrolled use of these insecticides can potentially affect the ecological environment, and almost all of them represent a threat to non-target organisms including humans [1]. In the last two decades, the use of neonicotinoids (neonics) spread worldwide, becoming the most important pesticide class currently present in [2] the global market as replacers of carbamates and organophosphates [3]. Neonic residues have been detected in fruits, vegetables, cereals, honey, drinking water, milk, soil, rivers, and bees [4,5,6]. Neonics mainly act against chewing and sucking parasites, predatory insects, zoophages, and phytophages as well as parasitic infection of pet animals as cats and dogs [7]. However, as neonics act on the nicotinic acetylcholine receptors of the targeted insect, they showed potential risks to mammalians including humans [8,9,10].

The negative effects of neonics on mammalian species include genotoxicity, cytotoxicity, immunotoxicity, neurotoxicity, reproductive disorders, hepatotoxicity, altered neuro-endocrine systems, and hormonal disorders [11].

Thiacloprid (TH) belongs to the neonics family and is widely used to provide a protection for various plants against a wide variety of insects. However, TH has also been shown to induce hepatotoxic, nephrotoxic, carcinogenic, and teratogenic effects in mammals and birds [12,13,14,15].

Chicoric acid (CA) is a natural dicaffeyltartaric phenolic compound found in various plant species such as *Echinacea purpurea*, *Cichorium intybus L*., *Cymodocea nodosa*, *Ocimum* *basilicum*, lettuce, dandelion, and iceberg [16]. It showed antioxidant, antiviral, and immunomodulatory anti-obesity activities [17] as well as a neuroprotective capacity owing to its ability to pass the blood–brain barrier and exert a strong scavenging activity [18].

Moreover, in vitro studies revealed that CA was also able to improve the viability of lipopolysaccharide-exposed BV2 microglial cells and glucosamine-exposed HepG2 cells [18,19,20].

Another natural compound with similar features to CA is rosmarinic acid (RA), which is an ester of caffeic acid and 2-hydroxy-dihydrocaffeic alcohol present in plants such as *Lamiaceae, Boraginaceae, Blechnaceae,* and *Zosteraceae* [21,22]. Additionally, RA showed antiviral, anti-inflammatory, antioxidant, anticancer, hepatoprotective, angiogenic, and antidepressant effects with various neuronal benefits such as protection against Kinate-induced convulsions and Parkinson’s disease [23,24,25,26,27,28,29]. Additionally, RA was shown to protect astrocytes and macrophages against apoptosis and reactive oxygen species (ROS)-mediated damages by enhancing brain antioxidants and reducing cytokines and inflammatory mediators [30]. Interestingly, RA also showed an attenuating effect on the morphine withdrawal syndrome via its opioid analgesic effect and potentiation of the GABA system [31,32].

In order to reduce animal testing and to predict the in vivo embryotoxicity without the influence of maternal factors, chicken embryos provide standard models for embryonic development to investigate the potential toxicity of various environmental pollutants [33].

The aim of this work was to assess the potential neurotoxicity of TH via investigating its oxidant, apoptotic, and inflammatory effects on the brain of chicken embryos and to evaluate the possible modulatory effects of CA and RA.

## 2. Materials and Methods

### 2.1. Chemicals

**Figure 1:**
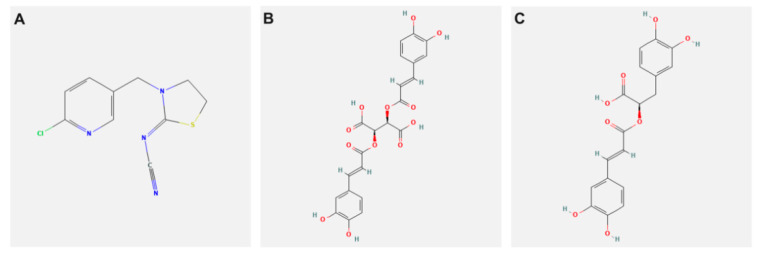
2D chemical structure representation of (**A**) thiacloprid, (**B**) chicoric, and (**C**) rosmarinic acid (PubChem source).

**Figure 2:**
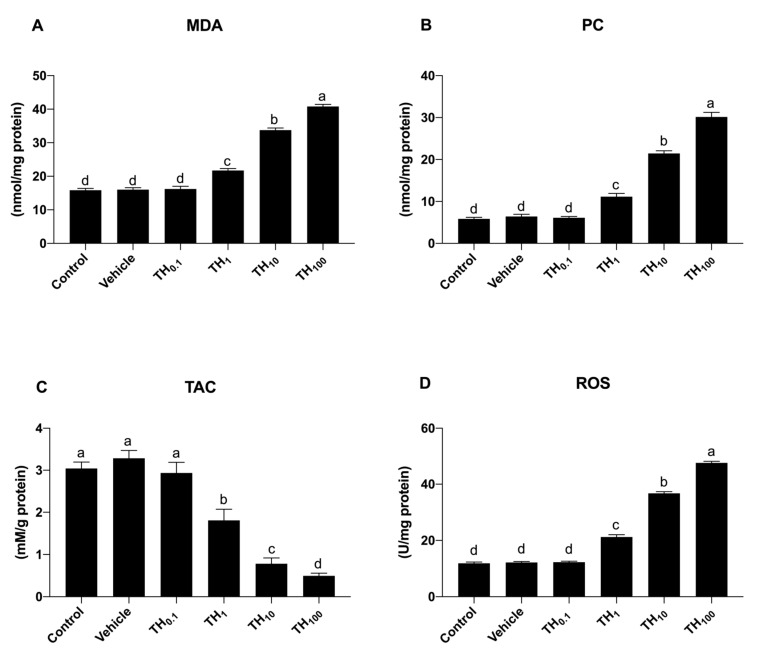
Schematic representation of the effect of TH on the oxidative stress markers in 19-day-old chicken embryos. (**A**) malondialdehyde (MDA); (**B**) protein carbonyl (PC); (**C**) total antioxidant capacity (TAC); (**D**), reactive oxygen species (ROS). Bars not sharing a common superscript letter (a,b,c,d) differ significantly at *p* < 0.05.

TH, [C_10_H_9_ClN_4_S;[3-(6-Chloro-3-pyridinylmethyl)-2-thiazolidinylidene]cyanamide (molecular weight: 252.7, CAS Number:111988-49-9, PESTANAL^®^, analytical standard] (Figure 1A). CA [purity ≥ 95%, C_22_H_18_O_12_ (2R,3R)-2,3-Bis {[(2E)-3-(3,4-dihydroxyphenyl)-1-oxo-2-propen-1-yl]oxy}butanedioic acid, 2,3-Di-trans-caffeoyltartaric acid (molecular weight: 474.37, CAS Number: 70831-56-0] (Figure 2A). RA [purity ≥ 98%, C_18_H_16_O_8_, (R)-O-(3,4-Dihydroxycinnamoyl)-3-(3,4-dihydroxyphenyl)lactic acid, 3,4-Dihydroxycinnamic acid (R)-1-carboxy-2-(3,4-dihydroxyphenyl) ethyl ester (molecular weight: 360.31, CAS Number: 20283-92-5] (Figure 1C). All chemicals were purchased from Sigma-Aldrich International GmbH (St. Louis, MO, USA).

### 2.2. Eggs and Birds

Fertilized chicken eggs (weighing 60 ± 5 g) were obtained from a commercial farm. Approximately (780 eggs) were used in two independent studies. The care of animals and experimental procedures were approved by Ethics of Animal Use in Research Committee (EAURC) at Zagazig University (ZU-IACUC/2/F/56/2021). Both the eggs and the hatching chicks were handled carefully and received the proper management, and unnecessary discomfort was avoided.

### 2.3. Experimental Design

#### 2.3.1. Dose-Response

##### Fertilized Egg and Management

The surface of the eggs was cleaned with povidone iodide and dried with clean dry tissue paper. The eggs were then candled in a dark room to discard broken and defective eggs (with a mean exclusion rate of 5%) and to mark the exact position of the air cell by a pencil. Three hundred and sixty cleaned fertilized eggs were used in this study and were randomly assigned to six equal groups of 60 eggs in triplicate. The groups were inoculated with TH at 0.1 (TH_0.1_), 1 (TH_1_), 10 (TH_10_), and 100 (TH_100_) µg/egg in 50 µL of a vehicle (sterile physiologic saline). The doses were selected to cover a wide range of environmental exposure and based on previous observations [13,14]. A fifth egg group was inoculated with equivalent vehicle volume (vehicle group). To evaluate the impact of the vehicle, a non-injected set of eggs was used (control group).

##### Air Cell Injections

The appropriate doses were inoculated into the eggs under sterile conditions on the third incubation day (the beginning of the embryogenesis process) to allow the translocation of TH into the entire body, including the developing brain. The broad end of the egg was cleaned using sterile gauze pads dipped in 70% alcohol solution. In the center of the egg surface, exactly over the air cell, a hole was drilled carefully using a sterile needle to avoid the shell membrane damage by the drill point. The needle was inserted horizontally into the air cell and wiped between each injection with a sterile gauze pad, and the shell holes were sealed with melted paraffin [34].

##### Chicken Embryo Incubation

After the inoculation, with the air sac up, the eggs were put in egg trays with holes to allow the flow of air around them. These trays were transferred to the incubator under standard conditions (temperature 37.8 °C, humidity 55%, and turned once/hour). Every other day, the eggs were candled, to detect dead or undeveloped embryos, which were regularly detected and excluded.

At the 19th incubation day, eggs were opened, embryos were carefully removed and separated from the yolk sac, and cleaned with PBS (phosphate buffer saline), and the brain was immediately taken out. Brain specimens were kept at −80 °C until analysis.

#### 2.3.2. Antidotal Study

This study was performed based on the results of the first experiment. Four hundred and twenty eggs were randomly assigned into seven treatment groups (in triplicate). The groups were the control group, TH (1 µg/egg), CA (100 µg/egg), and RA (100 µg/egg), each in 50 µL of the saline vehicle [0.85% NaCl (*w*/*v*) in water], TH/CA, TH/RA, and TH/CA + RA. The CA and RA doses were selected based on the results of a preliminary experiment performed in our laboratory. Management of the fertilized eggs, air cell injections, incubation of chicken embryos, embryos collection, and sampling of the brain all were carried out as previously illustrated in the first experiment.

##### Oxidative Injury Assays in Brain Tissue

The brain specimens were homogenized in cold PBS (pH 7.5) in 1:5 *w*/*v* ratios in an ice-cold water bath, in a Teflon Homogenizer, and then were centrifuged at 10,000 rpm (at 0–4 °C) for 15 min. Then the supernatant was used to estimate lipid peroxidation product (malondialdehyde; MDA) calorimetrically [35]. The protein carbonyl (PC) content was evaluated by using ELISA (MyBiosource, San Diego, CA, USA; Chicken ELISA kits; Catalog No. MBS735444). Total antioxidant capacity (TAC) was determined using Chicken Elisa (MyBiosource, San Diego, CA, USA; Catalog No. MBS9346021), while the reactive oxygen species (ROS) was determined using ELISA Kit (Signalway Antibody, College Park, MD, USA; Catalog No. EK20447).

##### Comet Assay

The oxidative DNA damage level in brains of the embryo models were determined using a comet assay following the method [36]. Fifty cells/slide were investigated and imaged by a CCD camera (Olympus, Tokyo, Japan) attached to the fluorescence microscope (Zeiss Axiovert Inc., Jena, Germany). Tail DNA% and tail length were determined for each cell. Moreover, Comet Assay Project software was used for estimation of the scores of tail moment from the comet image of each cell.

##### Inflammatory Response Markers

The inflammatory markers, including myeloperoxidase (MPO), nitric oxide (NO), interleukin–1β (IL-1β), and tumor necrosis factor-α (TNF-α), were determined using Chicken ELISA kits (MyBiosource, San Diego, CA, USA; Catalog No. MBS266621, MBS778196, MBS761055 and MBS165670, respectively).

##### Apoptotic Markers

Caspase 3 and Bcl-2 were determined using an Elisa kit (MyBiosource, San Diego, CA, USA; catalog No. MBS266210 and MBS73624, respectively). A microplate reader (Multiscan Ascent, Dasit s.p.a, Milan, Italy) was used for the measurement of markers’ concentration (450 nm absorbance).

##### Transcriptional Analysis of Stress and Inflammatory Cytokine-Related Genes in Brain

Total RNA was extracted from the frozen samples of brain by means of TRIzol reagent (easyREDTM, iNtRON Biotechnology, Korea). Then, the Quantitect^®^ Reverse Transcription kit (Qiagen, Germany) was used for the first-strand cDNA synthesis following the kits manufacturer’s protocol. The forward and reverse sequences of primers of the studied genes, Caspase-3, Bcl-2, interferon-γ (IFN-γ), interleukin-1β (IL-1β), tumor necrosis factor-α (TNF-α), and nuclear factor kappa-light-chain-enhancer of activated B cells (NF-κB) and the housekeeping β-actin gene, are reported in Table 1. Then, the qPCR analysis was performed using a Rotor-Gene Q instrument with a QuantiTect^®^ SYBR^®^ Green PCR kit (Qiagen, Germany) under the following thermocycler conditions: 10 min at 95 °C, followed by 40 cycles of 95 °C for 15 s and 60 °C for 30 s and 72 °C for 30 s. To verify the specificity of PCR, a melt-curve analysis was performed. The relative mRNA expression pattern for each gene was calculated using the comparative 2^−ΔΔCt^ method [36].

### 2.4. Statistical Analysis

Data were analyzed using GraphPad Prism 8 software (GraphPad Software, Inc., La Jolla, CA, USA) and reported as the mean ± standard error of the mean. Differences in oxidative stress biomarkers, DNA damage, inflammatory and apoptotic markers, and stress-related genes were analyzed using a one-way analysis of variance (ANOVA) followed by Tukey’s multiple tests. A value of *p* < 0.05 was considered statistically significant.

## 3. Results

### 3.1. Experiment 1: (Dose-Response)

#### Effect on Oxidative Stress Biomarkers

As far as concerns MDA, PC, and ROS, no significant differences between the control, vehicle, and TH_0.1_ groups were observed (Figure 2A,B,D), while a significant dose-dependent increase was observed following exposure to TH_1_ (*p* < 0.05), and this increase became more prominent both in the TH_10_ and TH_100_ group (*p* < 0.05) compared to the control, vehicle, and TH_0.1_, respectively.

Conversely, the TAC biomarker significantly decreased in a dose-dependent manner after the exposure to TH_1_, TH_10_, and TH_100_, respectively.

Because the oxidative stress biomarkers were modified starting from the exposure to TH1, this dose was chosen for more evaluation. Moreover, regardless of the marker, the results indicated no significant changes between the vehicle and control group, so the vehicle group was used as the control in the second experiment.

### 3.2. Experiment 2: (Antidotal Study)

#### 3.2.1. Effects on Mortality Rate and Oxidative Stress Variables

**Figure 3:**
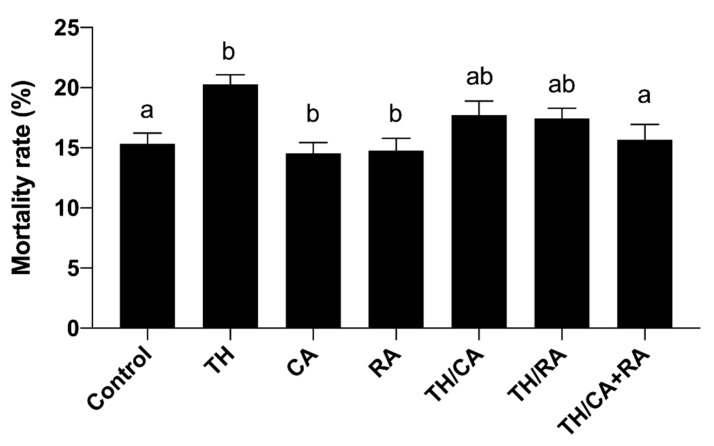
Effect of TH, RA, CA, and their combinations on the rate of mortality of exposed embryos. Bars not sharing a common superscript letter (a,b) differ significantly at *p* < 0.05.

Data in Figure 3 showed that the exposure of embryos to TH significantly increased the rate of mortality compared to the control. On the other hand, co-exposure to RA or CA improved the mortality rate, particularly in the TH/CA+RA group (*p* < 0.05).

Results also showed that brain tissue MDA and protein carbonyl contents were significantly higher in TH-treated embryos with respect to the control. MDA content was significantly low in all co-administered groups (TH/CA, TH/RA, and TH/CA+RA), where its level attained the control value. Additionally, protein carbonyl content increased up to the control value in the TH/CA + RA co-treated group but was still significantly higher in the TH/CA and TH/RA co-treated groups (Table 2).

The results showed that the TAC was normalized to control values in the TH/CA + RA group compared to control and other treatment groups. TH/RA induced a more significant improvement in the TAC than the TH/CA compared to the TH group. However, both treatments did not restore the TAC to the control value. The total ROS was significantly elevated after the TH exposure with respect to the control group, while TH/RA and TH/CA + RA treatments significantly modulated the total ROS level, which was comparable to the control value.

#### 3.2.2. Effects on DNA Damage

**Figure 4:**
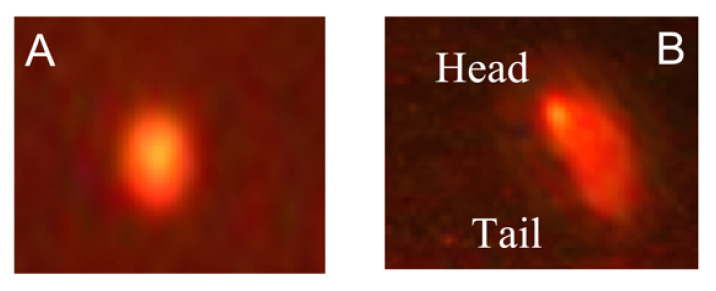
Comet images of cells derived from the brain tissues of embryos. (**A**) Normal contact cells derived from control, CA, and RA groups; (**B**) comet cells derived from TH-treated group.

The examination of the comet using fluorescence microscopy provided data about the DNA damage percentage represented by an elevation in the stained DNA migration length (Figure 4).

A significant increase in comet variables (tail length, tail DNA%, and tail moment) was observed in the brain of the TH-treated embryos with respect to control (*p* < 0.05).

**Figure 5:**
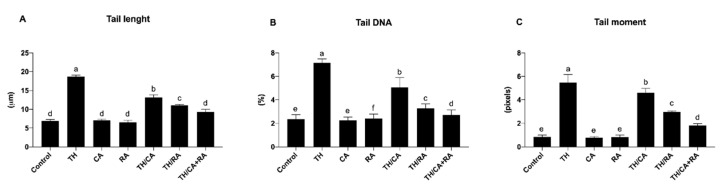
Schematic representation of the effect of TH, CA, and RA in ovo exposure on the comet variables: (**A**) tail length, (**B**) tail DNA %, and (**C**) tail moment in the brain of 19-day-old chicken embryos. Bars not sharing a common superscript letter (a–f) differ significantly at *p* < 0.05.

TH/CA, TH/RA, and TH/CA + RA significantly improved all DNA damage with respect to the TH-treated group (*p* < 0.05); in particular, the TH/CA + RA group of the tail DNA % was able to almost reach the same value of that of the control (Figure 5)

#### 3.2.3. Effects on Inflammatory Markers

The level of inflammatory response biomarkers (IL-1β, TNF-α, NO, and MPO) exhibited a significant enhancement in the brains of the TH-exposed embryos (*p* < 0.001). On the other hand, their levels were significantly improved in TH, CA, and/or RA co-exposed groups, where TH/CA + RA could restore them to control values (*p* < 0.001, Table 3).

#### 3.2.4. Effects on Apoptotic Markers

Table 3 shows that TH induced a significant increase in the level of Caspase-3 (Casp3) protein with respect to the control. The separate treatment with CA and RA equally decreased the TH-induced expression of apoptotic Casp3 protein, while their combined use showed a better reduction but still higher than the control. TH also significantly decreased the anti-apoptotic protein Bcl-2 with respect to the control. This reduction was significantly declined in both the TH/RA and TH/CA + RA groups, being comparable to the control value.

#### 3.2.5. Effect on Pro-Inflammatory Cytokines and Stress-Related Genes

**Figure 6:**
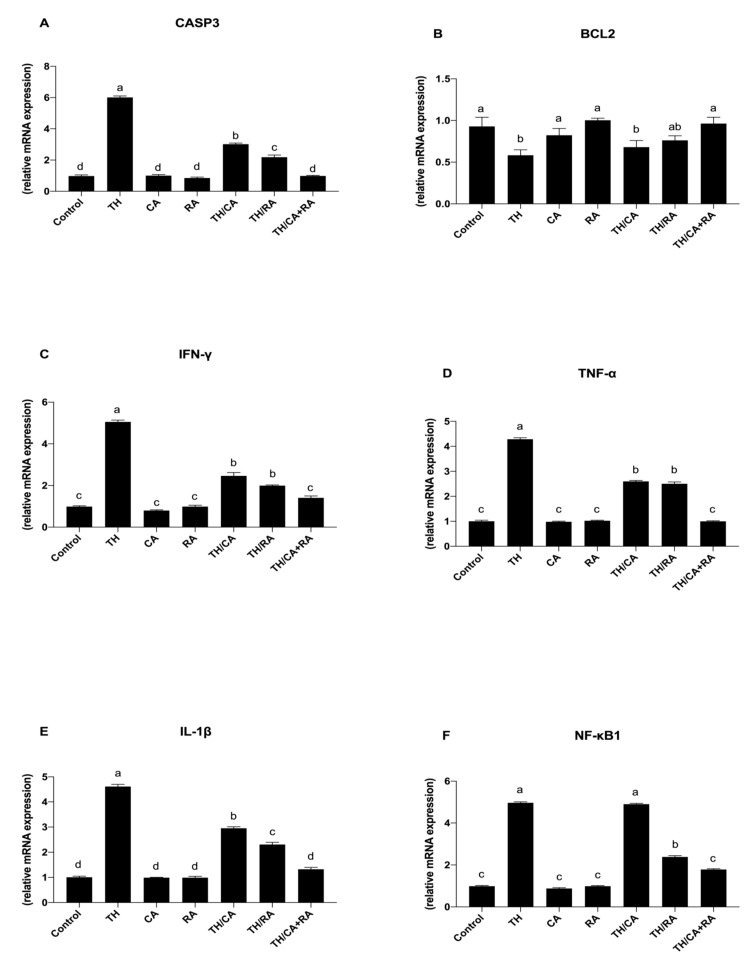
Schematic representation of the effect of TH, CA, and RA in ovo exposure on the expression pattern of (**A**,**B**) apoptotic-related genes (casp3, Bcl2) and (**C**–**F**) pro-inflammatory cytokines (INF-ƴ, TNF-α, IL-β, and NF-kB1) in the brains of 19-day-old chicken embryos. Bars not sharing a common superscript letter (a,b,c,d) differ significantly at *p* < 0.05.

TH-exposure induced a significant upregulation of the mRNA expression pattern of Casp3 in comparison to the control (*p* < 0.05) (Figure 6A). By exposure to TH/RA and TH/CA + RA, the pattern of this gene was significantly reduced compared to the TH-exposed group (*p* < 0.05), while the TH/CA + RA group significantly reduced the gene pattern up to the control one (*p* < 0.05).

An opposite trend was observed for the mRNA expression pattern of Bcl2 gene (Figure 6B).

Regarding the expression of pro-inflammatory cytokines and NF-KB, these genes were significantly upregulated in response to TH with respect to the control (*p* < 0.05) (Figure 6C–F).

Conversely, upon exposure to TH/CA and TH/RA, the expression profile of TNF-α and IL-1β genes was significantly reduced with respect to the TH group (*p* < 0.05) (Figure 6D,E), while a significant decrease with respect to TH was observed only after exposure to TH/RA for INF-γ and NF-KB genes (*p* < 0.05) (Figure 6C,F), returning to the control levels after exposure to TH/CA + RA both for pro-inflammatory cytokines and NF-KB genes.

## 4. Discussion

Chicken embryo presents morphological and molecular similarities to other vertebrates, particularly at the phylotypic period. The chicken eggs and embryos are easily handled and characterized by a quick development without the influences of the maternal factor. In addition, their higher sensitivity to the environmental pollutants makes them a suitable model for the assessment of the toxic impact on the development of vertebrates [34,37]. For these reasons, they were chosen in the current experiment.

Oxidative stress has been involved in the neurotoxic risk pathogenesis of numerous pesticides due to the high vulnerability of the brain to oxidative damage [38].

Therefore, in the current study, the biomarkers of oxidative stress were investigated as indicators of oxidative damage of cellular macromolecule as a possible mechanism of TH neurotoxic action. This study demonstrated that the brain TAC in TH-exposed embryos was significantly lowered. On the other hand, MDA and PC brain tissue content was significantly higher in TH-exposed embryos.

Additionally, the total ROS was significantly increased by TH treatment. These situations may induce impairments in the antioxidant mechanisms and metabolic detoxification of embryos.

The pesticide metabolism may produce oxidative molecules, which could alter the activity of antioxidant enzymes and the level of antioxidant biomarkers, leading to oxidative damage [39]. Oral administration of TH and imidacloprid increased the peroxidation of lipids and carbonyl contents in lymphoid organs, the plasma, kidneys, liver, and brain tissues of rats [12,40,41]. TH also caused oxidative stress in zebrafish (*Danio rerio*) [42] and embryos and larvae of common carp [43].

TH group showed excessive ROS production, which may reflect the failure of the antioxidant system to clean up the body from the ROS excess. Neonicotinoids showed the ability to increase the production of ROS via attacking the mitochondria and impairing its function [44]. When the level of ROS increases more than the antioxidant systems scavenging capacities, it results in lipids, proteins, and DNA oxidations [45,46,47,48]. This postulation is supported by the enhanced protein carbonyl formation, increased MDA production, and increased indices of DNA damage in the brains of TH-treated embryos. Aydin similarly detected significantly higher level of carbonylated proteins with decreased levels of antioxidant enzymes in rats after exposure to TH [41]. High protein carbonylation levels were also documented in *Eisenia fetida* [49] and *Prochilodus lineatus* [44] after clothianidin and imidacloprid treatments, respectively.

The alkaline comet assay is one of the excellent sensitive tests for determining the DNA damage and repair. The genotoxic potency of TH was shown in the TH-treated embryos, in which an elevation in the endpoints of comet test was observed, reflecting the extent of DNA damages, which could be mainly due to the increased ROS. The DNA oxidative damage was similarly reported in bovine peripheral lymphocytes [50], bovine cultures of whole blood [51], peripheral lymphocytes of humans [52,53], and rat bone marrow [12] after exposure to TH. DNA damage was also reported in acetamiprid-exposed CaCo-2 and imidacloprid-exposed HepG2 cells [54,55]. Similarly, significant elevations in the extent of DNA damages following the exposure to neonicotinoids were reported in *Danio rerio* and *Prochilodus lineatus* fish [44,56].

The DNA oxidation by xenobiotics may help the initiation of proapoptotic-encoding pathways resulting in cellular death, as the apoptosis process is mostly regulated by the cysteine proteases expressions, including caspases family members (the apoptotic pathways promoters) and the anti-apoptotic protein Bcl-2 [1].

The present study revealed that the level of Casp3 protein was significantly elevated in the TH-exposed group in comparison to the control. On the other hand, TH significantly reduced the Bcl-2 protein relative to the control. Furthermore, the transcriptomic data revealed upregulation of the caspase-3 expression pattern accompanied by downregulation of Bcl-2 mRNA expression in the brains of TH-exposed embryos as a response to cellular stress.

TH has been reported to induce cellular apoptosis in peripheral lymphocytes from humans [52], bovine peripheral lymphocytes [50], and zebrafish [42]. A similar observation was recorded in rat brain cells with the induction of DNA fragmentation and Caspase 3 activation [57] and in zebrafish Danio rerio [58] after imidacloprid treatment. High levels of apoptotic cells and significant apoptotic genes’ upregulation were reported with other pesticides such as diazinon, zineb, lambda cyhalothrin, and pirimicarb [35,59,60]. We suggested that TH-induced oxidative damage in the brain of embryos might explain its apoptotic action where ROS can also weaken the function of cerebral vasculature with consequent cell damages and death [61].

From the current findings, TH exposure resulted in oxidative damage in embryonic brain tissue, which consequently triggered or facilitated secondary inflammatory responses, likely to be mediated via the NF-κB signaling pathway. This was indicated by the obtained upregulation of TNF-α, IL-1β, and IFN-γ mRNA expression. The proinflammatory cytokines induction is a primary indicator of the inflammatory process [1]. NF-κB has a strategic position at the crossroad between inflammation and oxidative stress; it is suggested that ROS might act as a key secondary mediator responsible for the activation of NF-κB in response to various stimuli [62].

IL-1β is a pleiotropic cytokine that performs an essential function in regulating inflammatory and immune responses. It is one of the early expressed cytokines promoting a reaction cascade leading to inflammation [63]. INF-γ is a key mediator in CNS injury and neurodegenerative disorders. It participates in the brain damage by the expression of major histocompatibility complexes (MHC-I and II). TNF-α is involved in the early stage of inflammations. It activates lymphocytes and neutrophils, increases endothelial cell permeability, promotes the synthesis and release of many other cytokines, controls the metabolic activity of other tissues, and takes part in brain damage [64].

In the present study, the elevation of the proinflammatory mediators (INF-γ, IL-1β, TNF-α, and NF-κB) in the brains of TH-exposed embryos advocates the ability of TH to trigger the inflammatory process.

Furthermore, our findings showed that the oxidative damage induced by TH is ascribable to the elevation of NO levels, which in turn elevates the activity of MPO as it released into extracellular spaces during inflammation and the activation of neutrophils.

Additionally, MPO plays as a catalytic agent in the synthesis of hypochlorous acid, which has a toxic effect on the different cellular components, and this would increase the oxidative damage [65]. This could explain the parallel increase in NO and MPO in the current work.

The inflammatory responses upon exposure to TH were reported earlier by Aydin in polymorph nuclear cells and plasma of rats [41]. TH and imidacloprid were also to cause neurological disturbances in *Gallus domesticus* inducing negative effects on neurogenesis of developing embryonic brain, reducing cell proliferations, increasing apoptosis, and altering the histoarchitecture of brain tissue [66,67].

In the current study, CA and RA markedly offset the oxidative injury provoked by TH, demonstrating a mitigation of the oxidative burden. They also reduced the levels of DNA damage and inflammatory and apoptotic markers, reflecting the protective potentials of CA and RA against oxidative-mediated damages. However, the protective effect was more prominent in the embryos co-exposed to the both acids, indicating the presence of synergistic effect between them.

Chicoric acid was found to reduce lipid peroxidation and increase the antioxidants activity and TAC in liver of mice and prevent inflammations of hepatic cells associated with obesity via decreasing the IL-6, TNF-α, and MPO activity [68]. CA and its metabolites caftaric and caffeic acids could inhibit protein carbonylation and degradation induced by alkoxyl and hydroxyl radicals, suppress nitration triggered by hemin/nitrite/H_2_O_2_, suppress the decreased viability of lipopolysaccharide-exposed BV2 cells, and reduce the NO and ROS production [20]. Furthermore, CA supplementation ameliorated the apoptotic effect of H_2_O_2_ on SH-SY5Y cells through promoting the Keap1/Nrf2 pathways with a marked reduction in the inflammatory mediators levels (IL-1β, TNF-a, and MPO) and MDA and attenuated neuron damage, as indicated by histological observations in the hippocampus of mice brains [18]. The regulation mechanisms of CA may be attributed to its ability to induce cellular redox balance, reverse the mitochondrial dysfunction, decrease apoptosis of neurons, and inhibit the inflammation associated with the oxidative injury.

Several studies indicated the capability of CA to overcome the blood–brain barrier and perform a scavenging activity that, in turn, is able to enhance the brain antioxidant capacity [18]. The present experiment introduced scientific bases for utilizing the CA in nutrition of the developing embryos as a promising natural antioxidant.

The anti-inflammatory properties of CA have also been previously reported. CA reduced memory impairment and loss of neurons associated with lipopolysaccharides in C57BL/6J mice and downregulated glial overactivation by blocking the NF-kB and MAPK pathways. Therefore, CA can reduce the cytokines and inflammatory mediators regulated by NF-kB such as cyclooxygenase-2 (COX-2), iNOS, TNF-α, and IL-1β in BV2 microglial cells and mouse brain. The underlying anti-neuroinflammatory mechanism of CA is supposed to be exerted via blocking the NF-kB translocation and inhibiting the MAPKs and PI3K/Akt phosphorylation [20]. These results suggest that CA could have the ability to reduce neuro-inflammation and might be worth being investigated more deeply to evaluate its mechanistic roles in various neurodegenerative disorders.

Regarding RA, it attenuated H_2_O_2_-induced ROS production and apoptosis and modulated the upregulated Bax and the downregulated Bcl-2 in human SH-SY5Y cells.

Furthermore, RA stimulated the antioxidant heme oxygenase-1 (HO-1) enzyme in association with the signaling pathways of phosphatidylinositiol-3-kinase and protein kinase A [69].

Similarly, RA showed neuroprotective, antioxidant, and anti-inflammatory effects in epilepsy and neuropathic pain, resulting from chronic constriction of the sciatic nerve in rat models, and prevented Kinate-induced convulsion [70,71]. RA has been demonstrated to protect against Parkinson’s disease in mouse models and improved the motor function by reducing the production of mediators of inflammation and inhibiting the activations of microglia in ventral mid brain. Additionally, RA inhibited NF-κB nuclear expressions and downregulated the HMGB1, Myd88, and TLR4 expressions in cell and animal models of Parkinson’s disease [72]. RA was also demonstrated to exert antioxidant and anti-inflammatory actions in ischemic renal and hepatic tissues [73].

Essential oils of RA could prevent hepatic injury induced by carbon tetrachloride by scavenging the free radicals and balancing the levels of antioxidants such as catalase, glutathione peroxidase, and glutathione reductase enzymes [74]. Qiao et al. reported that lipopolysaccharide-induced iNOS and NO proteins were reduced by RA in RAW264.7 macrophages [75]. However, the underlying mechanisms of RA protective action still need more investigation. The obtained findings suggested that the anti-oxidative activity could possibly be a mechanism involved in the RA-mediated protections.

## 5. Conclusions

The findings of the current study showed that TH induced oxidative stress, inflammatory response, and the altered expressions of apoptotic and stress-encoding genes, indicating its neurotoxic impacts on the developing brain. CA and RA co-administered with TH showed a marked reduction of the toxic effect providing protection to the brain of developing embryos against TH toxicity, and their combined administration showed a synergistic action.

In light of these observations, the use of RA and CA is advisable due to their powerful natural antioxidant activity against neonicotinoids-induced oxidative injuries.

## Figures and Tables

**Table 1:** Primer sequences for studied genes used for RT-qPCR.

Gene	Sequences (5′-3′)	Accession Number
Caspase 3, apoptosis-related cysteine peptidase (Casp-3)	F: TGTGGACTCTGGAATTCTGCC R: AACGAGATGACAGTCCGGTA	NM_204725
B-cell CLL/lymphoma 2 (Bcl-2)	F: ATCGTCGCCTTCTTCGAGTT R: AGGCATCCCATCCTCCGTT	NM_205339
Interferon, gamma (IFN-γ)	F: GAACTGGACAGAGAGAAATGAGA R: ATGTGTTTGATGTGCGGCTT	NM_205149
Tumor necrosis factor-alpha (TNF-α)	F: TGCTGTTCTATGACCGCC R: CTTTCAGAGCATCAACGCA	AY765397
Interleukin-1beta (IL-1β)	F: CTACACCCGCTCACAGTCCT R: GCCTCACTTTCTGGCTGGA	NM_204524
Nuclear factor kappa (NF-κB1)	F: TACCGGGAACAACACCACTG R: CAGAGGGCCTTGTGACAGTA	NM_205134
Beta actin (β-actin)	F: CCCAAAGCCAACAGAGAGAA R: CCATCACCAGAGTCCATCAC	NM_205518

**Table 2:** Effect of TH, CA, and RA in ovo exposure on oxidative stress markers in brains of 19-day-old chicken embryos.

Parameters	Experimental Groups							*p*-Value
	Control	TH	CA	RA	TH/CA	TH/RA	TH/CA + RA	
MDA (nmol/mg protein)	16.29 ± 0.07 ^bcd^	21.81 ± 1.83 ^a^	14.18 ± 0.66 ^cd^	13.00 ± 0.57 ^d^	18.20 ± 0.14 ^b^	17.84 ± 0.20 ^b^	17.35 ± 0.62 ^bc^	<0.001
PC (nmol/mg protein)	6.05 ± 0.03 ^c^	12.44 ± 0.12 ^a^	5.14 ± 0.06 ^d^	5.00 ± 0.00 ^d^	8.23 ± 0.16 ^b^	7.73 ± 0.25 ^b^	6.71 ± 0.29 ^c^	<0.001
TAC (mM/g protein)	3.53 ± 0.01 ^ab^	1.46 ± 0.02 ^e^	3.61 ± 0.01 ^ab^	3.81 ± 0.08 ^a^	2.11 ± 0.00 ^d^	2.67 ± 0.14 ^c^	3.34 ± 0.12 ^b^	<0.001
ROS (U/mg protein)	12.56 ± 0.03 ^cde^	23.36 ± 2.88 ^a^	9.28 ± 0.53 ^de^	7.74 ± 0.23 ^e^	19.20 ± 0.47 ^ab^	17.38 ± 0.62 ^bc^	13.99 ± 0.38 ^cd^	<0.001

Values that are not sharing a common superscript letter (a,b,c,d,e) differ significantly at *p* < 0.05. Malondialdehyde (MDA), protein carbonyl (PC), total antioxidant capacity (TAC), and reactive oxygen species (ROS).

**Table 3:** Effect of TH, CA, and RA in ovo exposure inflammatory and apoptotic markers in brains of 19-day-old chicken embryos.

Parameters	Experimental Groups							*p*-Value
	Control	TH	CA	RA	TH/CA	TH/RA	TH/CA + RA	
Inflammatory Markers								
TNF-α (pg/mg protein)	59.80 ± 0.77 ^d^	93.58 ± 2.32 ^a^	64.36 ± 1.492 ^d^	47.58 ± 4.32 ^e^	80.23 ± 4.02 ^b^	72.15 ± 3.09 ^cd^	62.36 ± 1.72 ^d^	<0.001
IL-1β (pg/mg protein)	81.16 ± 0.55 ^d^	150.16 ± 1.15 ^a^	73.91 ± 2.71 ^e^	64.67 ± 1.09 ^f^	128.42 ± 3.17 ^b^	100.18 ± 0.04 ^c^	87.59 ± 1.45 ^d^	<0.001
NO (µmol/g protein)	25.17 ± 0.10 ^de^	51.51 ± 0.89 ^a^	21.95 ± 0.32 ^ef^	20.27 ± 0.19 ^f^	37.78 ± 1.58 ^b^	32.03 ± 0.69 ^c^	29.21 ± 01.97 ^cd^	<0.001
MPO (ng/mg protein)	1.07 ± 0.01 ^d^	12.74 ± 0.70 ^a^	1.20 ± 0.19 ^d^	1.15 ± 0.08 ^d^	7.24 ± 0.06 ^b^	3.81 ± 0.30 ^c^	1.37 ± 0.06 ^d^	<0.001
Apoptotic biomarkers								
Casp3 (ng/mg protein)	1.51 ± 0.00 ^d^	6.15 ± 0.02 ^a^	1.62 ± 0.11 ^d^	1.72 ± 0.07 ^d^	4.05 ± 0.33 ^b^	3.56 ± 0.02 ^b^	2.64 ± 0.20 ^c^	<0.001
Bcl-2 (ng/mg protein)	0.41 ± 0.00 ^b^	0.15 ± 00.00 ^d^	0.53 ± 0.02 ^a^	0.61 ± 0.03 ^a^	0.26 ± 0.03 ^cd^	0.30 ± 0.05 ^bc^	0.38 ± 0.01 ^bc^	<0.001

Values that are not sharing a common superscript letter (a–f) differ significantly at *p* < 0.05. Tumor necrosis factor-alpha (TNF-α), interleukin-1beta (IL-1β), nitric oxide (NO), myeloperoxidase (MPO), apoptosis-related cysteine peptidase (casp3), and B-cell CLL/lymphoma 2 (Bcl-2).

## Data Availability

The data presented in this study are available on request from the corresponding authors.
